# On Finding the Source of Human Energy: The Influence of Famous Quotations on Willpower

**DOI:** 10.5964/ejop.v13i4.1372

**Published:** 2017-11-30

**Authors:** Jesús Alcoba, Laura López

**Affiliations:** a International Graduate School, Centro Superior de Estudios Universitarios La Salle, Universidad Autónoma de Madrid, Madrid, Spain; Webster University Geneva, Geneva, Switzerland; The Maria Grzegorzewska University, Warsaw, Poland

**Keywords:** positive psychology, self-control, willpower, famous quotations

## Abstract

Positive psychology focuses on aspects that human beings can improve, thereby enhancing their growth and happiness. One of these aspects is willpower, a quality that has been demonstrated to have various benefits on people, as widely shown in the literature. As a result, a growing body of research is attempting to establish the conditions under which an individual’s willpower can be increased. This work attempts to confirm whether the famous quotations that people often use to inspire or motivate themselves can have a real effect on willpower. Two experiments were conducted assigning randomly subjects to a group and priming them with famous quotations, and afterwards comparing their performance in a willpower task with a control group. The second experiment added a willpower depletion task before priming. As a result, primed subjects endured the willpower task significantly more time than control group, demonstrating that famous quotations related to willpower help to increase this capacity and to counteract the effect of willpower depletion.

## Positive Psychology and Improvement of a Person’s Qualities

Although Psychology has traditionally focused primarily on the diagnosis and intervention in people’s psychological problems, a new current has emerged over recent decades, the goal of which is to study people who are not suffering from any psychopathologies in order to develop a theoretical and technical field that will help increase people’s degree of adjustment and happiness. The generic name for this approach is Positive psychology because, in principle, it does not focus on problems and negative issues, but rather on those aspects that people can improve, thereby increasing their personal growth and happiness (Seligman, 2011). Positive psychology interventions have demonstrated their capacity to improve people’s wellbeing (Sin & Lyubomirsky, 2009) and level of happiness (Proyer, Wellenzohn, Gander, & Ruch, 2015).

## Willpower

One of the areas that can be regarded as part of this general approach – given that it does not mainly focus on actions to treat any problematic Psychology, but rather seeks to improve human skills – is research into willpower.

The benefits of this capacity were observed decades ago by Mischel, who conducted a series of experiments showing that four-year-old children who were able to defer gratification for a longer time would later go on to develop into more cognitively and socially-competent adolescents, achieving greater academic performance and being better equipped to cope with frustration and stress (Mischel, Shoda, & Rodriguez, 1989).

These initial studies were confirmed years later in a longitudinal study that followed 1,000 children and 500 twins from birth to the age of 30, the results of which confirmed Mischel’s. It found that, irrespective of social class or intelligence, self-control alone could help to predict factors such as academic results, health, wealth, drug problems and even contact with the justice system (Moffitt et al., 2011).

It has also been described how greater self-control correlates with better academic results, a higher degree of psychological adjustment (lower level of psychopathology and greater self-esteem), fewer problems in controlling impulsive behaviour around food and alcohol, better relationships and interpersonal skills, secure attachment and better emotional responses (Tangney, Baumeister, & Boone, 2004).

However, despite its benefits, far from being a quality that is fully under human control, people make decisions that lead to immediate gratification even if these will be damaging in in future, instead of taking other decisions that, although they will not bring any short-term pleasure, will in fact benefit them more. This behaviour occurs even in cases where people are quite sure they will be sorry for making this decision afterwards (Magen & Gross, 2007).

Precisely through observing these faults in the self-regulation process and by conducting a series of initial experiments (Baumeister, Muraven, & Tice, 2000), a line of research has appeared that attempts to look into these phenomena in more depth. The results have been broadly popularised in the publication *Willpower* (Baumeister & Tierney, 2012). According to this model, self-control is a function that is necessary for success and which acts as a limited resource. In other words, as a person carries out certain actions, their reserve of willpower is drained (ego depletion). These actions fall into the following types – controlling thoughts, managing emotions, resisting impulses, focusing attention, channeling behavior and making decisions (Baumeister, Vohs, & Tice, 2007). Exercising any one of these reduces self-control in all of them. In other words, there is no independent reserve of willpower for each task and, conversely, exercising self-control in one area increases this capacity in all the others (Baumeister et al., 2007). These considerations have led the authors in this field of research to state that self-control functions in a similar way to a muscle (Muraven & Baumeister, 2000) and that, in the same way, it can recover through rest (Tyler & Burns, 2008). This model has been tested in the areas of food, drink, spending, sexual behavior, intelligent thought, decision-making and interpersonal conduct (Baumeister et al., 2007). Although this field of research is broad and there are still many aspects that require clarification, it has been globally validated by a recent meta-analysis (Hagger, Wood, Stiff, & Chatzisarantis, 2010).

These ideas have led to the emergence of a growing body of research that aims to determine the conditions under which the depletion of willpower can be counteracted. There is known to be a relationship between glucose and willpower, because it has been demonstrated that acts of self-control reduce the levels of this substance in the blood, and that consuming glucose counteracts the depletion of willpower caused by carrying out a task when then undertaking another that requires this capacity (Gailliot et al., 2007).

However, regardless of these findings, a series of studies has shown that there are other factors, without such a direct biological link (unlike the consumption of glucose, or rest), which also have an effect on the recovery of willpower. For example, a study by Schmeichel and Vohs (2009) showed that reaffirming a person’s fundamental values counteracts the effect of willpower depletion, suggesting that the key factor here is the greater level of mental engagement involved in reflecting on these values. Another study has shown that activating positive emotions (through subliminal priming) also manages to counteract the effect of self-control depletion (Ren, Hu, Zhang, & Huang, 2010). Research has also been conducted into the relationship between self-awareness and self-control, showing that an increase in self-awareness also helps to restore self-control in people who have previously suffered from depletion of this capacity (Alberts, Martijn, & de Vries, 2011). Another area of study has researched the way in which optimism impacts on the depletion of self-control, finding that activating an optimistic outlook (through priming) helps to counteract the effect of self-control depletion in subjects with dispositional optimism, but not in others (In Den Bosch-Meevissen, Peters, & Alberts, 2014). Following the accumulation of all this evidence, the question that now emerges is how to practically facilitate the replenishment of self-control beyond the limitations of experimental research, in which subjects are typically placed in a controlled situation and given explicit instructions.

Humans interact with the world through representational models that mediate between their actions and reality. Language plays a fundamental role in these models, because it contributes to cognitive development, facilitating the establishment of analogies and relationships and providing a representational system that aids abstract thought (Gentner, 2003). Following these principles, this study seeks to establish whether the use of famous quotations has a role to play on willpower. These well-known phrases tend to be of value in that they are written in a beautiful way, or because they have been uttered by historical figures with whom a person wants to identify. It is therefore common to find them used in advertising slogans or shared on social media, and people amass them as pearls of wisdom. However, to date, no study has investigated their real potential to bring about change, or to help people achieve their goals. This study was designed with the intention of finding out the effect of these famous quotations on willpower, and comprises two experiments. The hypothesis of the first experiment was that priming people with the famous quotations related to willpower would increase this capacity among a group of subjects compared with a control group. In the second experiment both groups underwent ego depletion by taking part in a tedious task, in order to see whether the famous quotations were able to significantly replenish willpower levels among the group primed with these phrases.

## Procedure and Results

### Experiment 1

19 college students in the field of health (17 females and 2 males) took part in this study, having been selected by a person unaware of the study’s objectives. The students were also unaware of the study´s objectives. The subjects were randomly assigned to two experimental conditions, the first with 10 subjects and the second with 9, as set out below:

- Primed. These subjects were asked to imagine that they had to deal with a difficult situation, such as a competitive exam for a civil service job, or a very hard physical trial, and they were given a sheet of paper with a set of 9 famous quotations relating to willpower (example: "I've failed over and over and over again in my life and that is why I succeed." - Michael Jordan), from which they had to choose the three they felt would help them most in this situation. They then had to copy these phrases on the back of the sheet. All this information was written in a document.
- Not primed. While the primed group were doing this, the non-primed group members were given a sheet of paper with 9 neutral phrases to copy on the back of the sheet (example: “They say that tomorrow morning it will rain in mountain areas, and it will be very cold. It may also snow in the higher areas.”). These instructions were also written in a document.

Next, all the participants had to solve a set of difficult anagrams, some of which were in fact impossible. The time that each subject took to give up on the task was recorded as a measurement of their willpower. Subjects from the primed group (the ones who had copied the famous quotations) were expected to keep trying for longer to solve the anagrams.

As it can be seen in Table 1, the primed subjects spent longer trying to solve the anagrams. The Kruskal-Wallis test was carried out to test whether this difference was relevant. As it can be seen, the test detected a significant difference. In other words, the mere fact of having copied three famous quotations on the back of a sheet of paper substantially increased the participants’ willpower, expressed as the time taken to give up on an impossible task.

**Table 1:** Results Experiment 1

Experimental condition				Kruskal-Wallis	
Not primed		Primed			
*Mdn* (sec)	*SD*	*Mdn* (sec)	*SD*	*z*	*p*
758	170	1109	124	-3.021	.001

### Experiment 2

Experiment 1 showed that willpower related famous quotations could improve willpower. However, a different angle about this phenomenon is related to whether those quotations can counteract willpower depletion. In order to test this, another experiment was conducted. In this second experiment, a further condition was added to Experiment 1, precisely to test whether, as well as increasing willpower among a group of subjects, the famous quotations would be able to recover their willpower after being depleted.

Some 38 college students in the field of health (12 males and 26 females) took part in this study, having been selected by a person unaware of the study’s objectives. The subjects were randomly assigned to two experimental conditions, each with 19 subjects.

Firstly, all the subjects carried out a test in which they had to cross out all the letters “a” in a neutral text (the *lorem ipsum* type, commonly used in provisional document layouts) for 10 minutes. In the following test, the two experimental conditions were added:

- Depleted-primed. As in Experiment 1 these subjects were asked to imagine that they had to deal with a difficult situation and they were given a sheet of paper with the same set of 9 famous quotations to do with willpower, from which they had to choose the three that they felt would help them most in this situation. They had to copy these phrases on the back of the sheet. This test took 10 minutes.
- Depleted-Not primed. As in Experiment 1, while the primed group were doing this, the non-primed group were given a sheet of paper with the same 9 neutral phrases to copy on the back of the sheet, listing them according to a numerical order that appeared at the start of each phrase.

Next, just as in Experiment 1, all the participants had to solve a series of very difficult anagrams, some of which were impossible. Once again, the time taken for each subject to give up on the task was recorded as a measurement of their willpower. It was expected that the depleted subjects in the primed group (who had prioritized the famous quotations) would keep going longer trying to work out the anagrams.

Before confirming whether there were significant differences between the groups taking part in the experiment, a test was done to check whether the depletion resulting from the crossing-out task had had the desired effect. In order to check whether the differences between the times invested in the two experiments, a comparison was made between the medians, which can be seen in Table 2. As it can be seen, there are significant differences between the two primed groups and the groups that were not primed, respectively.

**Table 2:** Comparison of Medians Between the Groups in the Two Experiments

Priming condition	Exp. 1 (not depleted)		Exp. 2 (depleted)		Kruskal-Wallis	
	*Mdn* (sec)	*SD*	*Mdn* (sec)	*SD*	*z*	*p*
Primed	1109	124	713	335	-2.501	.011
Not primed	758	170	530	124	-3.665	< .001

This experiment focuses on whether the famous quotations can counteract the effect of willpower depletion. For this reason it was relevant to find out if the designed task (crossing out all the letters “a” in a text) actually depleted subjects’ willpower. The way to verify this was to analyze if the subjects of the second experiment (both those who had been primed and those who had not) invested more time in trying to solve the anagrams, as it was the case.

With regard to the difference between the groups participating in the second experiment, Table 2 shows that the subjects in the “Depleted-primed” experimental group spent a longer amount of time working on the anagram test, suggesting that the famous quotations increased their willpower.

To check whether the difference observed was statistically significant, after conducting a test that confirmed the normality assumption (Shapiro-Wilk), a t-test was calculated. Since there is no homoscedasticity of variances between both groups (Levene´s test), the corrected value of “*t*” for these circumstances was obtained and so it was verified that the difference is statistically significant (Table 3).

**Table 3:** t-test Experiment 2

Experimental condition	Levene’s test		*t*-test	
	*F*	*p*	*t*	*p*
Depleted – primed vs. depleted - not primed	18.144	< .001	2.363	.027

To sum up, simply prioritizing a series of famous quotations for 10 minutes was enough to increase the willpower in primed subjects, offsetting the depletion caused by the first test.

## Discussion and Conclusions

Humans interact with the world through representational models that mediate between their actions and reality, and language plays a fundamental role in these models (Gentner, 2003). The two experiments described in this study show that priming subjects with famous quotations related to willpower helps to increase this capacity and to counteract the effect of willpower depletion. This demonstrates the important effect of language and cognition, not just on people’s mental lives, but also on their capacities. In fact, it has been shown that people’s ability to carry out a task increases when they conduct a cognitive reconstruction of it as a test of their willpower (Magen & Gross, 2007).

One possible explanation for the results obtained in these experiments is that the phrases used magnify fundamental human values. It is known that thinking about core values has the power to increase reserves of willpower and prevent their depletion (Schmeichel & Vohs, 2009), and that personal values can increase performance, as demonstrated in the academic environment, although subsequent studies will be needed to study this relationship in greater depth and clarify it (Parks & Guay, 2012).

Other explanations for these results should be sought in alternative explanations for the model of self-control set out in this study. In particular, it has been proposed that motivation and positive emotions could be factors involved in reducing or replenishing a human being’s willpower (Hagger et al., 2010). In the particular case of the experiments carried out here, it is possible that the famous quotations may have acted either to generate motivation or to induce a positive state of mind able to restore the subjects’ reserves of self-control. In this respect, the results of both experiments are consistent with preceding research relating the increase in willpower with fundamental values (Schmeichel & Vohs, 2009), the activation of positive emotions (Ren et al., 2010), self-awareness (Alberts et al., 2011) and optimism (In Den Bosch-Meevissen et al., 2014). In this sense, there is a broad field of research with respect to the relationship between the use of language as a way of representing the world, the emotions that it can elicit and willpower. From this perspective, more research is needed to clarify how these variables are dynamically related in order to draw conclusions that facilitate the understanding of human behavior and also to derive practical recommendations.

Although the results obtained are statistically significant, this study also has various limitations. Firstly, all the subjects were university students, and most of them were women. This makes it difficult to draw any conclusions about the effect of similar studies on other types of samples. Secondly, in the first experiment the number of subjects in the sample used was significantly smaller, meaning that non-parametric tests had to be used. Thirdly, the subjects of the study – as with any group in the general population – had different levels of willpower (Hagger et al., 2010) and as this variable was not monitored (because there is no way to measure willpower without depleting it at the same time) it is not possible to precisely determine how the depletion of self-control reserves and subsequent replenishment of these reserves impacts on subjects with higher or lower levels of willpower at the outset. A fourth limitation is that the mere imagination of the difficult situation could have influenced willpower, even before working with the famous quotations. Lastly, but by no means least importantly, the study does not take into account the effects of the results obtained over the medium or long term.

This study joins a growing body of research that, within the general area of Positive psychology, enables people who are not suffering from any psychopathology to further develop their own capacities, in this case self-control, which is related, as described, with a series of benefits ranging from better academic results and better psychological adjustment to fewer problems with controlling impulsive behavior around food and alcohol, improved relationships and interpersonal skills, secure attachment and better emotional responses (Tangney et al., 2004). In this respect, as a recommendation, it is considered relevant to incorporate the ideas emanating from this study into those actions in which willpower is an important variable, such as psychological or academic intervention programs. For example, the use in those interventions of famous motivational quotations that are aligned with the values of subjects participating in such programs.

From these reflections it is to be hoped that more studies like this one will be carried out in future, and that they will continue to offer new avenues for human personal development.
